# A Higher Frequency Administration of the Nontoxic Cycloartane-Type Triterpene Argentatin A Improved Its Anti-Tumor Activity

**DOI:** 10.3390/molecules25081780

**Published:** 2020-04-14

**Authors:** Zaira Tavarez-Santamaría, Nadia J. Jacobo-Herrera, Leticia Rocha-Zavaleta, Alejandro Zentella-Dehesa, Beatriz del Carmen Couder-García, Mariano Martínez-Vázquez

**Affiliations:** 1 Departamento de Productos Naturales. Instituto de Química, Universidad Nacional Autónoma de México, Mexico City, Circuito Exterior s/n, Ciudad Universitaria, Mexico City 04510, Mexico; zaira_03@live.com.mx (Z.T.-S.); betty-couder@hotmail.com (B.d.C.C.-G.); 2 Unidad de Bioquímica. Instituto Nacional de Ciencias Médicas y Nutrición Salvador Zubirán, Vasco de Quiroga 15, Sección XVI, Mexico City 14000, Mexico; nadia.jacobo@gmail.com (N.J.J.-H.); azentell@iibiomedicas.unam.mx (A.Z.-D.); 3 Departamento de Biología Molecular y Biotecnología, Instituto de Investigaciones Biomédicas, Universidad Nacional Autónoma de México, Circuito Mario de la Cueva s/n, Ciudad Universitaria, C.P. Mexico City 04510, Mexico; lrochaz@biomedicas.unam.mx; 4 Departamento de Médicina Genómica y Tóxicología Ambiental & Programa Institucional de Cáncer de Mama, Instituto de Investigaciones Biomédicas, Universidad Nacional Autónoma de México, Circuito Deportivo s/n, Ciudad Universitaria, Mexico City 04510, Mexico

**Keywords:** Argentatin A, colon cancer, cell senescence, xenografts, antiproliferative, apoptosis, antitumor, PCNA

## Abstract

*Parthenium argentatum* (Gray), commonly known as guayule, has been used to obtain natural rubber since the beginning of the 20th century. Additionally, the so called “resin” is a waste product derived from the industrial process. The cycloartane-type triterpene Argentatin A (AA) is one of the main constituents of the industrial waste resin. In this study we evaluated the AA anticancer activity both in vitro and in vivo in the HCT116 colon cancer cells. The apoptosis promotion of AA was assessed by the annexin V/propidium iodide (PI) assay. The senescence was evaluated for SA-β-galactosidase, and PCNA was used as a marker of proliferation. Its antitumor activity was evaluated using a xenograft mouse model. The results indicated that AA-induced apoptosis in HCT-116 cells and was positively stained for SA-β-galactosidase. In the xenografted mice test, the administration of AA at the dose of 250 mg/kg three times a week for 21 days reduced tumor growth by 78.1%. A comparable tumor reduction was achieved with cisplatin at the dose of 2 mg/kg administered three times a week for 21 days. However, nude mice treated with AA did not lose weight, as they did remarkably when treated with cisplatin. Furthermore, the animals treated with AA showed similar blood profiles as the healthy control group. These data indicate the low toxicity of AA compared to that shown by cisplatin.

## 1. Introduction

Cancer continues to be a disease of global concern [1]. Worldwide, colorectal cancer ranks second in death by cancer for both sexes and all ages and is the thirst most common neoplasia [2]. Because of the complexity of this illness, its treatment is far from optimal. However, in the last two decades the overall survival rate has doubled, reaching an estimated median survival expectancy of 20 months. The latter is largely due to new therapeutic approaches that rely on the combined administration of novel molecular agents with well-known cytotoxic agents, or drug repositioning [3]. The former are modern agents first approved for medical use between the first decade of 2000 (e.g., bevacizumab, panitumumab, cetuximab, aflibercept, and regorafenib), whereas the second have a longer history of use (e.g., oxaliplatin, irinotecan, 5-fluorouracil, capecitabine), with only a few being more modern (e.g., trifluridine/tipiracil) [4,5]. However, the toxicity of these therapies remains a critical limitation, justifying the search for new treatments [5].

The secondary metabolites produced by plant species or their derivatives are among the several experimental approaches for the development of new strategies to treat colorectal cancer [6]. The triterpenes belong to a group of secondary plant metabolites that are distinguished by the fundamental backbone of a 30-carbon isoprenoid molecule and have come to the attention of researchers because of their various biological properties especially those related to cancer treatment [7,8]. Through a significant amount of preclinical studies in tumor xenograft models, the chemopreventive and therapeutic effects of naturally occurring triterpenes and synthetic derivatives have been demonstrated, with a particular low general toxicity [9]. For instance, the ability of the betulinic acid, a pentacyclic triterpene, to take part in a central event of the apoptotic process by means of triggering the mitochondrial membrane permeabilization has been linked to its antitumor properties [10]. Likewise, the anti-inflammatory effect of betulinic acid has been demonstrated in vivo in a mice model of carrageenan-induced pleural inflammation [11]. The absence of toxicity was reported within a dose of 30 mg/kg of betulinic acid, and a protection to cadmium chloride-induced toxicity was observed in a dose-dependent manner [12].

The so-called argentatins A and B are the main cycloartane-type triterpenes isolated from the resin obtained as a side product of the natural rubber production process from *P. argentatum* (Gray), commonly referred as “guayule”, which means “plant that contains rubber”, a native desert flowering shrub distributed from northern Mexico to the Southwestern United States [13,14]. Our own studies, as well as other research groups, have documented that for each kilogram of rubber produced, one kilogram of resin is obtained. The content of the argentatins A and B in the resin is 10%, making them very attractive as industrial supplies [15].

As revealed through numerous epidemiological studies, chronic inflammation contributes to the predisposition of different types of cancers [16]. Our previous research showed that argentatins A and B have anti-inflammatory activity in a TPA (12-O-tetradecanoyl-phorbol-13-acetate) edema-induction model [17,18]. We have also reported both the cytotoxic activity against various human cancer cell lines and the antitumor effect in a xenograft prostate model of Argentatin B (AB) from doses as high as 500 mg/kg [19]. Considering the chemical similarity between AB and AA we hypothesized that AA could also have anti-cancer activity. To test this hypothesis we evaluated the anti-tumor effect of AA in xenotransplants of human colon cancer cells. 

## 2. Results

### 2.1. Cytotoxicity In Vitro of AA on Cancer Colon Cells

**Figure 1:**
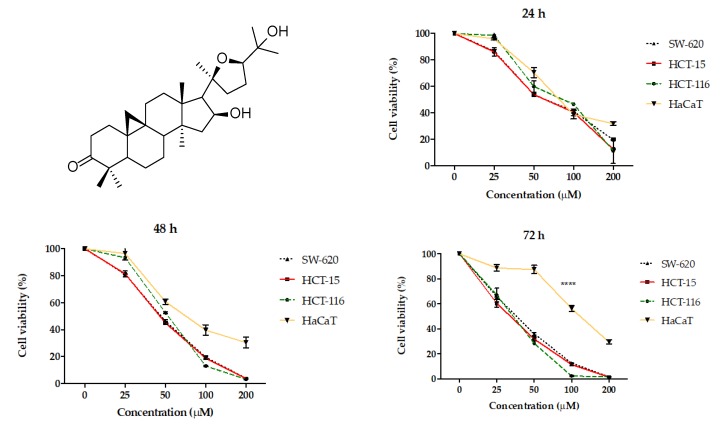
Chemical structure of Argentatin A (AA) and graphs of cell viability in terms of percentage with respect to concentrations of AA in µM. The lines in the graphs represent the standard deviation average of 3 independent experiments performed in triplicate each ± SD. **** *p* < 0.0001 by *t*-test.

As previously reported, AA (Figure 1) was isolated from guayule resin and its physical and spectroscopic properties (melting point, ^1^H and ^13^C, Nuclear Magnetic Resonance) were compared to those reported in the literature for identification purposes [13,14,15]. The crystal violet method was used to test the cytotoxic effect of AA in the human colon cancer cell lines (HCT15, HCT116, and SW620) and normal epidermal keratinocytes cell line (HaCaT). The cells were subjected to concentration ranges of 25 to 200 μM of AA (Figure 1). Our results showed that AA induced dose and time dependent cytotoxic activity against colon cancer cells. The results showed that HCT116 and HCT15 were equally chemosensitive, while the SW620 cell line displayed resistance (Figure 1 and Table 1). By means of comparison with a non-tumor cell line, the HaCaT cells were exposed to AA. The IC_50_ of 121.45 ± 1.3 is indicative of the AA being at least five times less cytotoxic in inhibitory concentration values for 72 h of treatment compared to the effect of cisplatin (22.26 ± 0.4 to 72 h). (Table 1).

### 2.2. Apoptosis Assay

**Figure 2:**
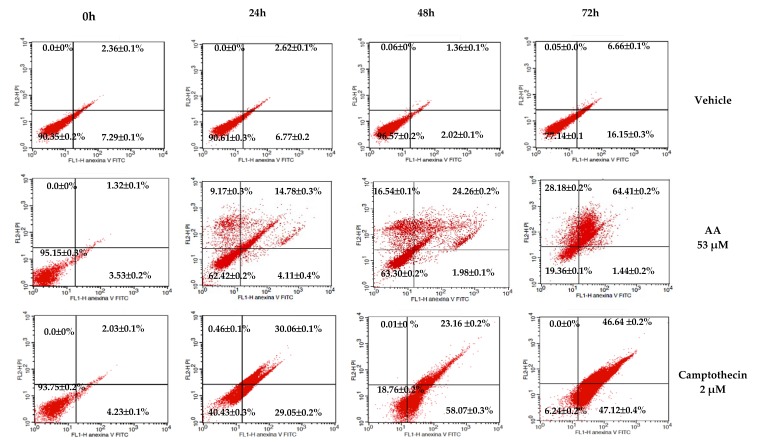
In vitro evaluation of induction of AA apoptosis in the HCT-116 colon cancer cell line using annexin V/PI. Representative dot plots of the HCT-116 cells treated with 53 µM of AA or with 2 μM of camptothecin at 0, 24, 48, and 72 h. The image acquisition was performed at 20× on the microscopic scale in the capture software ProgRes CapturePro. The numbers in the quadrants represent the average of 3 independent experiments ± SD.

Apoptosis induction by AA in HCT-116 cells was assayed by double staining with annexin V/PI. Our results showed that AA induced 64.41% apoptosis at 72 h and 53 μM (Figure 2). Camptothecin was used as a positive control.

### 2.3. Evaluation of β-Galactosidase Activity on HCT-116 Cells Treated with AA

**Figure 3:**
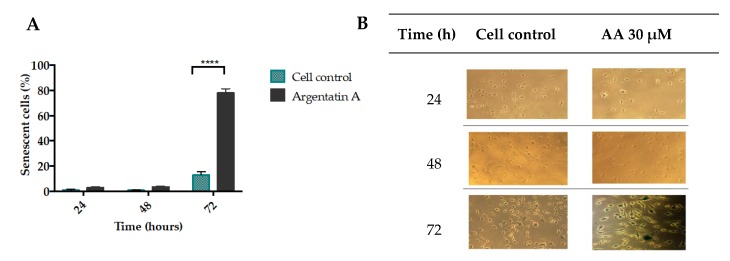
Evaluation of senescence induction of AA in the HCT-116 cell line. (**A**) Graph of senescence induction where the percentage of SA-β-galactosidase induced by treatment with AA (30 μM) in the HCT-116 cell line at 24, 48, and 72 h is indicated. The bars represent the average ± SD of 3 independent experiments, **** *p* < 0.0001 (*t* test) significant difference compared to the control. (**B**) Representative photomicrographs of the HCT-116 cell line stained with SA-β-Galactosidase Staining Kit, which were treated with AA (30 μM) at 24, 48, and 72 h, image acquisition was performed at 20× on the scale microscopic in ProgRes CapturePro capture software.

As observed in Figure 3A,B, AA induced almost 80% of senescence cells activity of galactosidase in HCT-116 cells at 72 h and 30 μM.

### 2.4. Toxicity of AA In Vivo

**Figure 4:**
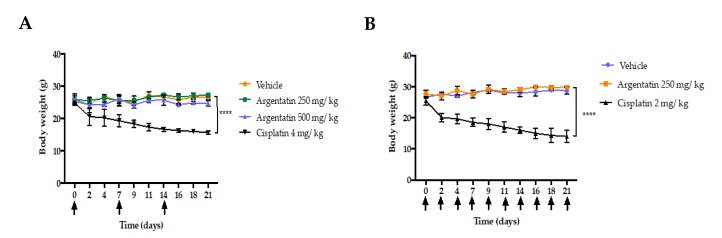
Effect of AA on the bodyweight of mice. (**A**) The mice received AA (250 or 500 mg/kg), cisplatin (4 mg/kg), or vehicle (10% DMSO in sesame oil) once weekly for three weeks on the days indicated with the black arrows. The mice were humanely sacrificed on day 21. (**B**) The mice received AA (250 mg/kg), cisplatin (2 mg/kg), or vehicle (10% DMSO in sesame oil) three times a week for three weeks. The days of administration showed with the black arrows. The mice were humanely sacrificed on day 21. Data are shown as average ± SD with n = 6 animals for each experimental group. **** *p* < 0.0001 stands for the statistical difference in the change in body weight between the beginning and the end of the experiment.

The administration of AA at 125, 250, or 500 mg/kg doses once a week for 21 days did not show toxicity or loss of body weight in *nu*/*nu* mice. However, those treated either with cisplatin at 4 mg/kg weekly for three weeks, or 2 mg/kg three times a week for three weeks, showed a significant body weight loss (Figure 4A,B).

Still, mice treated with AA either at 500 mg/kg once weekly or 250 mg/kg three times a week did not show behavioral or physical changes in comparison with those of the control group. However, mice treated with cisplatin either at 2 mg/kg three times a week or 4 mg/kg once weekly showed an increase in hepatic toxicity and a decrease in leukocytes (Table 2).

### 2.5. AA Inhibited Tumor Growth

**Figure 5:**
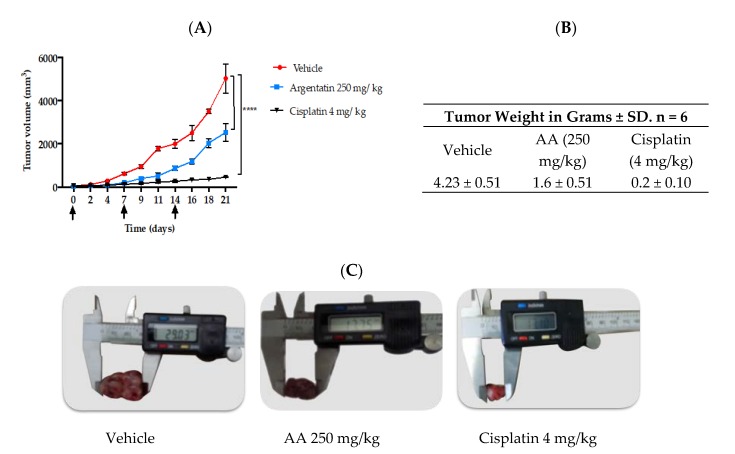
In vivo antitumor effect of administration of AA once weekly for 21 days. The antitumor activities of AA were evaluated in *nu*/*nu* xenotransplanted mice with human colon cancer cells (HCT116). (**A**) Graph of the tumor volume in mm^3^ against the days of treatment, the black arrows indicate the days of administration with AA, cisplatin or vehicle. Plotting symbols represent the mean ± SD of six mice for each experimental group. **** *p* < 0.0001, significant differences compared to the negative control. Data were analyzed using ANOVA and *t* test. All treated groups were statistically different from the negative groups. (**B**) Tumor weights ± SD at the end of the experiment. (**C**) Representative photographs of tumor size at the end of the experiment.

**Figure 6:**
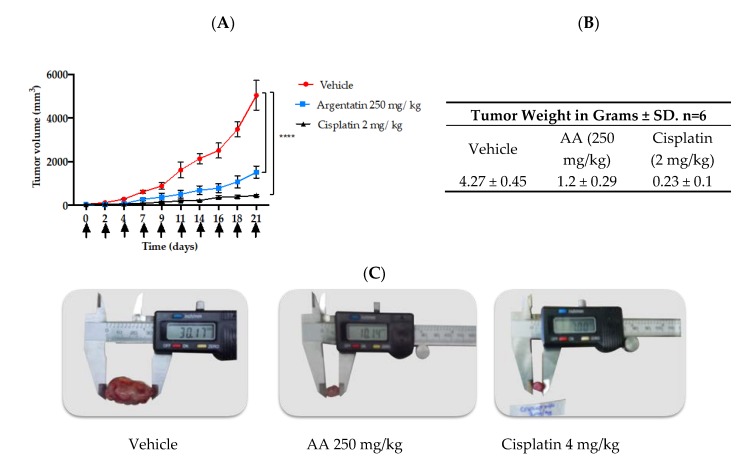
In vivo antitumor effect of AA. The antitumor activities of AA were evaluated in *nu*/*nu* xenotransplanted mice with human colon cancer cells (HCT116). (**A**) Graph of the tumor volume in mm^3^ against the days of treatment, the black arrows indicate the days of administration with AA, cisplatin or vehicle. Plotting symbols represent the mean ± SD of six mice for each experimental group. **** *p* < 0.0001, significant differences compared to the negative control. Data were analyzed using ANOVA and *t* test. All treated groups were statistically different from the negative groups. (**B**) Tables of tumor weights ± SD. (**C**) Representative photographs of tumor size.

The treatment schemes for xenotransplated mice with HCT116 are depicted in Figure 4A,B. Treatment began when xenotrasplants reached 50 mm^3^. The administration of AA at 250 mg/kg once weekly for three weeks reduced tumor growth in 49.19% (Figure 5A). However, the administration of AA at 250 mg/kg three times a week for three weeks was more effective than administered once weekly. The results showed a reduction of 78.14% concerning those untreated mice (Figure 6A).

When the experiment ended the tumors of each group were removed and photographs of the tumors were taken (Figure 5C and Figure 6C). Notably, in Figure 5C the largest diameter was always that of the negative control groups with 29 mm, however, the group treated with AA had a final diameter of 17 mm. The tumor size of the cisplatin-treated groups was smaller (13 mm). In Figure 6C it is observed that the largest diameter was always that of the negative control groups with 30 mm compared to the average diameter of the AA-treated groups (10 mm) and the cisplatin-treated groups (7 mm), but, there was no significant difference between the tumors of the groups treated with AA at 250 mg/kg and the cisplatin group at 2 mg/kg.

After removal, the tumors were weighed and compared to each other. In both schemes, the most significant tumor weight was that of the negative control group (approximately 4 g). Similarly, in both schemes, the cisplatin-treated groups achieved the lowest weight on average 0.2 g, from the group treated with AA 250 mg/kg three times a week for three weeks, tumors of lower weight on average 1.2 g were obtained (Figure 5B and Figure 6B).

### 2.6. Histological and Immunohistochemical Analysis of Tumor Tissues

**Figure 7:**
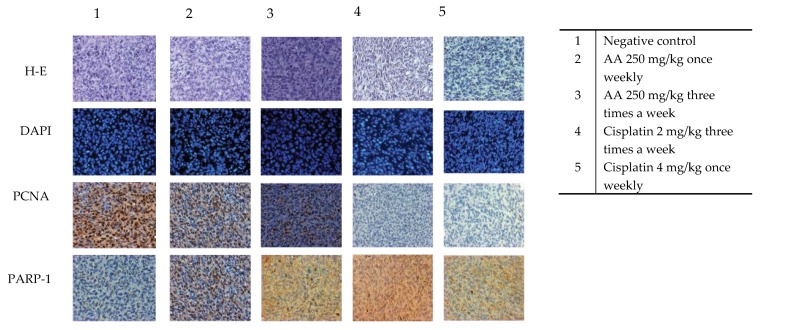
Representative photomicrographs of tissues acquired from xenotransplanted tumors with HCT-116 cells, stained with DAPI, HE, specific immunostaining for PCNA and PARP-1. Images were acquired using a 20× microscopic scale and the Q capture pro 5 QImaging capture software.

To evaluate whether the administration of AA could induce changes in cellular morphology in the tumor tissues, hematoxylin-eosin staining was performed. The results showed that tissues treated with AA had elongated nuclei and more space between them, which suggests an expansion of the cytoplasm area. These results suggest that the cells of the treated tissues are suffering some damage compared to the control group (Figure 7).

DAPI (4-6-diamidino-2-phenylindole) was used for the recognition of fragmented nuclei associated to cell death. As can be observed in Figure 7, the treated groups and the control group are very similar, there are no fragmented nuclei that could be indicating cell death. However, it is to be noted that the cells of the groups treated with AA were more elongated and enlarged (Figure 7).

**Figure 8:**
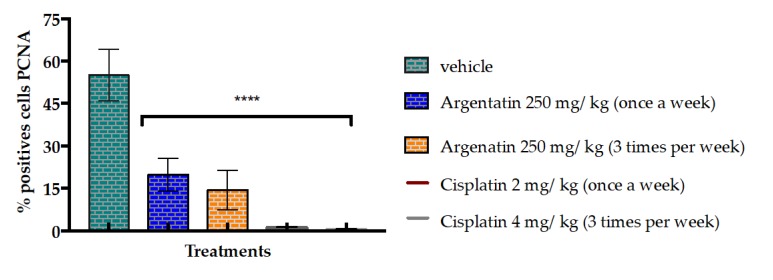
Graph of the evaluation of the antiproliferative effect of AA on the PCNA cell proliferation marker in the tissues of xenotransplanted tumors. The bars represent the average ± SD of the positive cells of three tissues analyzed in each group. The data were analyzed and compared against the negative control **** *p* < 0.0001 using *t* test.

The inhibition of cell proliferation induced by AA was evaluated by the expression of PCNA (Proliferating Cell Nuclear Antigen), a marker of cell proliferation. Administration of AA at 250 mg/kg dose three times a week for three weeks decreased the average number of cells positive for PCNA (Figure 7). The percentage of the treatments with AA ranges from 10%–20%, while the percentage of cells positive for PCNA in the control group was 54.81% ± 15.63. These results, together with data obtained from the photomicrographs, showed that there is less proliferation of cells in the groups treated with AA at different schemes of treatments, compared with those of the control group (Figure 8).

**Figure 9:**
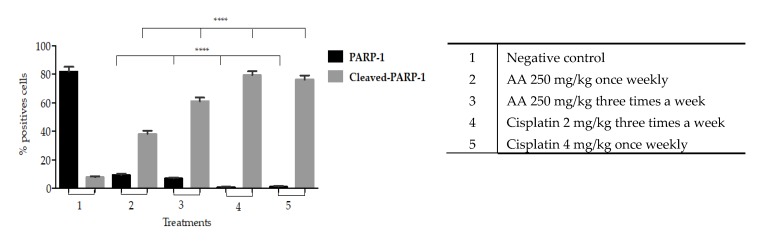
The graph shows the apoptotic cell death effect by AA on the levels of PARP-1 and cleaved-PARP-1 marker in the histological sections of xenografted tumors of HCT116 cells. The bars represent the average ± SD of the positive cells of three tissues analyzed in each group. The data were analyzed and compared against the negative control **** *p* < 0.0001 using *t* test.

The specific immune staining of anti-PARP-1 (Figure 7) was used to determine the induction of apoptosis response in histological sections. The results showed that the level of PARP-1 in the nucleus under any schemes of doses of AA is significantly lower compared to the vehicle (81.87% ± 3.15) (Figure 9).

The levels of cleaved PARP-1 were significantly increased with the dose of 250 mg/kg (38.24% ± 2.03) once weekly, and 250 mg/kg three times a week (61.03% ± 2.60) compared to the vehicle (7.71% ± 0.76). Treatments with AA always resulted in lower percentages of cleaved-PARP-1 compared to cisplatin doses (*p* < 0.0001) (Figure 9).

### 2.7. Western Blot Analysis

**Figure 10:**
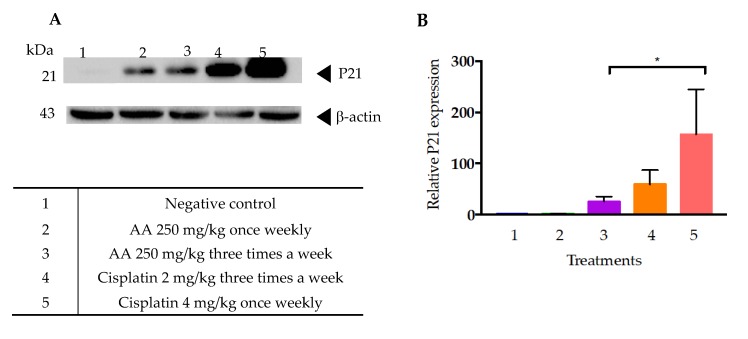
AA increases the expression of proteins associated with cellular senescence. (**A**) Western blot analysis of the expression of the marker of the negative regulator of the cell cycle p21 in response to the treatments with AA, cisplatin, and vehicle from the extraction of proteins from the tumor by each experimental group. (**B**) Graph of the relative expression of the bands obtained in Western blot. The bars represent the average ± SD of the positive cells of three tissues analyzed in each group. Data were analyzed and compared against the negative control * *p* < 0.0001 by *t* test.

The Figure 10A,B show the western blot transfer analysis of cell lysate from the tumors obtained from the xenotransplanted mice with the HCT116 cell line, which were treated with AA and cisplatin. Apparently, there was no expression of p21 in the administration scheme of treatments given once weekly. However, when the dose and frequency of AA was increased an overexpression of the p21 protein was clearly observed. Treatments with cisplatin led to 3-fold increased expression of p21, compared to the treatment with AA, as can be seen in the graph (Figure 10B).

## 3. Discussion

Our research group published the anti-inflammatory activity and the moderate cytotoxicity of the cycloartenol-type triterpene AB in different cancer cell lines, and its antitumor activity in vivo [17,18]. In a xenograft model of HCT15 and PC3, the AB decreased the growth rate of the tumors in nude mice, in doses as high as 500 mg/kg [19]. Such results were valid and promising despite the NCI guidelines for anticancer activity of plant extracts and pure compounds [20]. Then, we rationalized that given the structural similarity between AA and AB triterpenes and based on these results, it was proposed that AA could then act as an antitumor agent in a xenotransplant model of colon cancer.

To select the colorectal cancer line with greater AA sensitivity, the HCT116, HCT15, and SW620 lines were tested. The results showed that the chemosensitive to AA of HCT116 and HCT15 were equivalent while the SW620 cell line displayed resistance. A notable fact is that AA did not show such a deep cytotoxic activity in the non-cancerous line HaCaT, this effect was more noticeable at 72 h. However, this line was attacked by cisplatin in the same way as cancer lines (Figure 1 and Table 1). In this regard, it is notable to consider that there is a greater cytotoxic effect for the HaCaT cell line with the use of cisplatin compared to AA at 72 h.

In the apoptosis test (Figure 2), AA exerts a higher toxic effect (64.41%) after 72 h of treatment in the HCT116 cell line. This result suggests that the HCT116 cells are more sensitive to the effect of AA and that the effect is time-dependent. For that reason, we selected this cell line to perform the rest of the experiments. 

The molecular mechanisms and signaling pathways that drive cell senescence have been previously reported [21]. However, the loss of the proliferation-competent cells’ ability to divide has turned out to be one key factor since it distinguishes senescent cells from those in the reversible state of quiescence, and from mature and thus terminally differentiated cells. In this sense, it has been suggested that cycle protein inhibitors (e.g., p21 and p16), a diminished DNA replication capability, and a reduced level of PCNA contribute to cell senescence [22]. The most common and thus widely used marker of senescence is the lysosomal senescence-associated β-galactosidase [23]. Our results showed that AA inhibited cell proliferation and induced almost 80% of senescence-associated β-galactosidase HCT-116 positive cells (Figure 3). 

The low toxicity of AA was demonstrated by the fact that the administration of this triterpene at 125, 250, or 500 mg/kg doses once weekly did not show toxicity or loss of body weight in *nu*/*nu* mice. In contrast, those treated either with cisplatin at 4 mg/kg once weekly for three weeks, or 2 mg/kg three times a week for 21 days, showed a significant body weight loss (Figure 4A,B). Although AA shows a low cytotoxicity profile in vitro, its in vivo activity was clearly evident. 

Although some physiological disorders can be correlated because of toxicity processes. It is also true that the toxic effects induced by certain xenobiotics can be seen in a blood test.

Liver enzymes are commonly found in liver cells and when the liver is damaged, liver cells release their enzymes into the blood stream; thus, increased levels of these enzymes are a symptom of liver damage. Aminotransferases ALT and AST are the most sensitive liver enzyme. On the other hand, acute kidney injury (AKI) is defined by a rapid increase in serum creatinine, decrease in urine output, or both. The administration of AA at 250 or 500 mg/kg doses during the time of experimentation did not induce damage to the liver (no increase in transferases) or kidney (no increase of creatinine), as did cisplatin (Table 2). Another experimental fact indicating the low or no toxicity of AA is that the administration of this triterpene did not induce loss of body weight as did cisplatin. Taking all these experimental facts into account, we can postulate, that despite the high dose of AA used in the in vivo experiments. AA in the experimental conditions used is not toxic or that its toxicity is low enough not to be detected.

In vivo tests of new antitumor agents are often carried out using daily administrations of the drug for short periods, however our study showed that 3 administrations of AA per week were enough to have an antiproliferative effect. Notably, in the in vivo experiment the AA was more effective as the frequency of administration dose increased, however this was true only at lower doses (Figure 5 and Figure 6). Toxicity of cisplatin (4 mg/kg) was made evident by the loss of body weight (Figure 4A). Furthermore, mice treated with cisplatin also presented leukopenia, lymphopenia and high aspartate transferase and alanine transferase levels at both 2 mg/kg and 4 mg/kg dose, as well as a significant increase in urea and creatinine serum levels, consistent with previous reports (Table 2) [24].

A remarkable difference between previously published activities against HCT-116 cells and that showed by AA is that AA induces senescence in HCT-116 cells. It appears that senescence induction is characteristic of AA and AB. Both AA and AB induced senescence in the HCT-15 cancer lines [19]. 

Senescence, as well as genomic instability, telomere attrition, low proteostasis, and impaired intercellular communication, are some of the cellular and molecular characteristics of cellular aging. Betulinic acid, a triterpene widely distributed in nature with low cytotoxic activity against cancer cells, has been reported to damage the membranes of the mitochondria and lysosome in no-cancer HaCaT cells, thereby initiating abnormal autophagy processes leading to the induction of replicative senescence and cell death [25]. 

Similar to betulinic acid, the argentatins A and B showed modest cytotoxic activity against some cancer lines suggesting that this activity is not the primary mechanism of the antitumor activities of these triterpenes. However, according to our results, the induction of senescence by argentatins both in vitro and in vivo against cancer cell lines would indicate that this process could be one of the main antitumor mechanisms of argentatins.

An important characteristic is that senescence in vitro is a phenomenon not as fast as cytotoxicity. In some cases, detection of senescence was achieved after 18 days of treatment [26], detection of senescence after 96 h of usage with the test agent has also been reported [25].

In our case the senescence was detected after 72 h after treatment. 

To the best our knowledge this is the first time that a senescence inducer triterpene is evaluated against HCT-116 xenograft in mice. Therefore to select the best doses to evaluate in our xenograft experiment, we taking into account the doses of argentatin B used against HCT-15 and PC-3 xenograft assay, where 500 mg/kg were even used once a week [19], we decided to evaluate AA at a dose of 250 mg/kg three times a week. Although it could be assumed that these doses are remarkably high compared to cytotoxic agents, our results indicate that they are not toxic to test animals.

The AA senescence induction in the xenograft tumors was evident by DAPI (4-6-diamidino-2-phenylindole) stained. As can be observed in Figure 7, the treated groups and the control group are remarkably similar, there are no fragmented nuclei that could be indicating cell death. However, it is to be noted that the cells of the groups treated with AA were more elongated and enlarged. One characteristic feature of senescent cells is flat, enlarged and heterogeneous cell shapes [27]. 

Another evidence is that the administration of AA at 250 mg/kg dose three times a week for three weeks decreased the average number of cells positive for PCNA. It has been reported that an excessive level of cyclin D1 represses cell proliferation by inhibiting DNA replication and cdk2 activity through the binding of cyclin D1 to PCNA and cdk2, as it does in senescent cells [28]. 

In addition, it has already been mentioned that cellular senescence is associated with higher β galactosidase (SA-β-gal) activity levels, and with the activation of several cell cycle arrest regulators, mainly p53-p21 and p16-Rb [29]. In this regard, it is notable that the cells of tumors treated with cisplatin, both at the dose of 2 and 4 mg/kg, showed a higher expression of p21 than those treated with AA (Figure 10). This data suggests that cisplatin could also induce a process of cellular senescence in xenotransplanted tumors with the HCT-116 cell line. In this regard, a study by Qu K et al. [30] showed that cisplatin was able to induce irreversible inhibition of proliferation and arrest of the G1 phase of liver cancer cells HepG2. Also reported were elevated levels of β-galactosidase-associated senescence in HepG2 cells subjected to low doses of cisplatin, as well as increased levels of TP53 and p21 expression [30].

On the other hand, our results show that there is a significant increase in cleaved-PARP1 into the cytoplasm induced by AA treatment that indicates cell death due to apoptosis. This effect was more evident in the histological tissues of xenotransplants treated three times a week compared to those treated once weekly (Figure 9). 

Expression of p21 is usually linked to p53 and DNA injury [31], at this point the mechanism by which AA-treatment leads to expression of p21 remains unknown. However, the increase in apoptosis upon treatment could be related to DNA damage.

It has been reported that cancellation of senescence or apoptosis is a prerequisite for tumor formation. Even more, the efficacy of anticancer agents also depends on the activation of apoptosis or an acutely inducible form of cellular senescence called “premature senescence” [32].

In the war against cancer, traditional therapeutic strategies have focused on looking for cytotoxic effects on the premise that the destruction of tumors will optimize the patient’s survival potential. However, although these approaches may lead to the disappearance of a solid tumor, they can also cause serious side effects in patients [33], thereby diminishing drug security. In addition, it is well known that these approaches often end up promoting drug resistance as well [33]. 

Aiming to underpin the potential and advantages of natural compounds and adjuvant therapies often excluded from in vivo studies, our research group decided to challenge the idea that only aggressive cytotoxic sources can be used in antitumoral therapies. Our research strategy was built around the need to develop friendlier, yet effective, methods to accompany the fight against tumor development. The research involved a challenging optimal dose selection of AA capable of producing the desired effect while also keeping a safe profile. 

Much discussion has arisen around the usefulness of studying natural products as antitumor agents [6]. Although attention has been drawn to the subject, big pharma is generally not interested in studying natural agents because said agents do not appear to have patent potential. On the other hand, the cytotoxic effects of natural drugs such as AA are often seen as negligible when compared to traditional or novel molecular agents. However, the fact that the industry is not generally interested, does not mean that natural drugs lack potential to be developed as new therapeutic agents or adjuvants.

Our results clearly indicate that induction of senescence by Argentatin A slows down the rate of tumor formation in vivo. Clearly in this approach the toxicity of the evaluated compounds is not as important as in other models. This peculiarity allows apparently higher doses to be administered compared to cytotoxic compounds.

These results indicate the possibility of combining AA with another drug with a different mechanism of action, for example a cytotoxic one. In such case, we could hypothesize that AA would induce premature senescence which in consequence enhance the activity of the second drug.

## 4. Materials and Methods 

AA was isolated from the resin as previously reported [13,14,15]. The resin was generated as a side product during the process of industrialization of the species *Parthenium argentatum* (Gray), and was donated by the National Commission of Arid Zones located in Saltillo, Coahuila, Mexico. 

### 4.1. Cell Lines and Cell Culture

Human colon carcinoma cell lines (HCT-116, HCT15, SW-620) were cultured in Dulbecco’s Modified Eagle Medium (DMEM), supplemented with fetal bovine serum (FBS 10%) and antibiotics (100 IU/mL of penicillin and 100 g/mL of streptomycin). Cultures were kept under controlled conditions of temperature (37 °C), CO_2_ atmosphere (5%) and relative humidity (95%). Normal epidermal keratinocytes cell line (HaCaT) was cultured under the same conditions. 

### 4.2. Solutions

Solutions of 25 mg/mL of AA in DMSO were prepared for the in vitro experiments by serial dilutions of 20, 40, 80, and 200 μM of AA in 2% FBS-supplemented DMEM, keeping a final DMSO concentration below 0.2%. Concentrated solutions of 25 mg/mL dissolved in 5% DMSO and sesame oil were prepared for the in vivo experiments. 

### 4.3. Cytotoxicity Assay

HCT15, HCT116, SW620, and HaCaT cell lines, separately seeded in 48-well culture plates at a density of 5 × 10^4^ cells per cm^2^ in 2% FBS-supplemented DMEM, were treated with serial dilutions of 25, 50, 100, and 200 μM (based on previous reports [17,18,19]) of AA after a 24 h incubation period. Cell viability was assayed after 24, 48, and 72 h of treatment. For the positive control, cisplatin was used at serial dilutions of 5, 10, 20, and 40 μM. For the negative control group, cells were grown in 2% FBS-supplemented DMEM with 0.2% DMSO. After incubation with the different treatments, the cells that remained adhered to the wells were fixed with a solution of 1.1% glutaraldehyde. The fixation medium was later removed and stained with 200 μL of crystal violet for 15 min. Excess of crystal violet was then washed off and crystal violet finally absorbed by the protein was solubilized in 10% acetic acid. A microplate reader at a wavelength of 595 nm was used to read the optical density values.

### 4.4. Apoptosis Determination

Colon cancer cell line HCT-116, seeded in 6-well plates at a density of 1.5 × 10^5^ cells per well, were incubated for 24 h and afterwards treated with vehicle, AA (53 μM) and camptothecin as positive control (2 μM) for different time periods (24, 48, and 72 h). The cells were then collected with trypsin and washed with PBS. After centrifugation at 1500 rpm for 5 min, the cell pellet was re-suspended in binding buffer and Annexin V-FITC, and propidium iodide was added following the recommendations of GeneTex Annexin V-FITC Apoptosis detection kit. A FACSCan flow cytometer from the National Flow Cytometry Laboratory at the National Autonomous University of Mexico (UNAM) was used to analyze the cell samples. A total of 10,000 cells were analyzed using the BD CellQuest Pro Software.

### 4.5. Cytochemical Staining with SA-β-Galactosidase

2 × 10^3^ cells per well were seeded in 24-well plates and incubated with 30 μM of AA at 24, 48, and 72 h. Cytochemical staining was performed with SA-β-Galactosidase Staining Kit at pH 6.0 using the Kit instructions (#9860, Cell Signaling). At the suggested time the medium was removed, and the wells were washed with PBS, after which a 1x fixative solution was added. After 10–15 min the wells were washed twice with 500 μL of PBS and the plate was later incubated at 37 °C for 14–16 h in a free CO_2_ chamber and a 1× maintenance solution was added, previously adjusted to pH 6.0. All experiments were performed in triplicate. The cell count was performed in a Nikon T-MSF clear field microscope. Blue stained cells were considered positive for SA-β-Galactosidase. The wells were divided in 0.4 cm^2^ squares, and all cells were counted and normalized to the average of cells per square.

### 4.6. Toxicity Test in Nu/Nu Mice

To test the toxicity of AA, 6-week-old female mice, whose weight ranged between 20 and 27 g, where bundled in groups of three. AA was administered intraperitoneally (i.p.) in two treatments schemes:

- Once weekly regimen for three weeks (21 days). Doses of 250 and 500 mg/kg of AA were administered i.p. and compared to the treatment of 4 mg/kg of cisplatin.
- Three times a week for three weeks (21 days). The dose of 250 mg/kg AA was administered i.p. and compared to the treatment of 2 mg/kg of cisplatin (in previous toxicity tests of our own, mice would not survive when cisplatin was administered at a dose of 4 mg/kg three times a week)

The weight and behavioral responses were recorded daily throughout the treatment and observed for 7 days following the last injection. Hematological analyzes were performed in the Department of Pathology of Veterinary Medicine and Zootechnics at UNAM.

### 4.7. Xenotransplant Assay

*Scheme 1: once weekly for three weeks.* 4–6 week-old male *nu*/*nu* mice were distributed in 3 groups of 6 mice each. The mice were acquired in the Bioterium of the National Institute of Medical Sciences and Nutrition Salvador Zubiran (INCMNSZ) in Mexico City. The mice were kept in Micro-Isolator^®^ cages in a pathogen-free environment, fed ad libitum and kept at room temperature of 22 °C, relative humidity of 55% and dark light cycles of 12/12 h. Approval by the Animal Research Committee was granted before performing the experimental procedures, which were carried out in accordance with the Guidelines for the Care and Use of Laboratory Animals of INCMNSZ (the institutional registration code BQO-1488-15/17-1). The mice were implanted with 1.5 × 10^6^ HCT-116 cells. The cells were re-suspended in 0.1 mL of PBS for subcutaneous inoculation in the right flank of the animal’s back. The treatments initiated once the tumors reached a volume of approximately 50 mm^3^ (Day 0). 

The mice received i.p. treatment once weekly for 3 weeks (days of administration: 0, 7, and 14). The study groups were as follows:

- 250 mg/kg of AA
- Positive control: 4 mg/kg of Cisplatin
- Negative control: 10% DMSO and sesame oil extra virgin Inés^®^

*Scheme 2: three times a week for three weeks.* 4–6 week-old male *nu*/*nu* mice were distributed in 3 groups of 6 mice each. Mice care was performed as in Scheme 1. The mice were implanted with 1.5 × 10^6^ HCT-116 cells. The cells were inoculated as previously described. The treatments initiated once the tumors reached a volume of approximately 50 mm^3^ (Day 0).

The mice received i.p. treatment three times a week for three weeks (days of administration: 0, 2, 4, 7, 9, 11, 14, 16, 18). The study groups were as follows:

- 250 mg/kg of AA
- Positive control: 2 mg/kg of Cisplatin
- Negative control: 10% DMSO and sesame oil extra virgin Inés^®^

The mice in both schemes were weighed daily. The tumor was measured with a calibrator (Vernier digital). The tumor volume was calculated using the formula V = π/6 × (larger diameter × [smaller diameter]^2^). The experiment lasted 21 days, after which the animals were humanely sacrificed and the tumor was removed, preserved in 10% formaldehyde and embedded in paraffin for future immunohistochemical test.

### 4.8. Hematoxylin-Eosin (HE)

The experimental sequence is summarized in the following steps. Mice were humanely sacrificed after treatment. The tumor tissues were sectioned into 4 μm-thick sections for immunohistochemical analyses (IHC). The deparaffinization consisted in exposing the tissues to a temperature of 60 °C for 15–30 min, followed by rehydration. The samples were put in contact for 5 min with the following solutions: xilol (x2), ethanol/xylol 50:50, ethanol 100%, ethanol 96%, ethanol 80%, ethanol 70%, ethanol 50%, distilled water, and PBS. A solution of Triton x-100 0.5% was used to permeabilize the cell membrane (30 min), followed by 2 washes with PBS of 5 min each. Hematoxylin 100–200 μL was added to cover the sample and distilled water was used to wash the samples. Samples then were covered with eosin and finally assembled with EcoMount from Biocare Medical. Photomicrographs were taken from xenotransplant tissues of HCT116 cells stained with HE. We acquired 30 photomicrographs per experimental group. The acquisition of images was done through an Olympus IX71 inverted microscope using the Qcapturepro 5 software of the company QImaging at 20× of the microscopic scale.

### 4.9. 4-6-Diamidino-2-Phenylindole (DAPI)

After removing the paraffin as previously described, DAPI staining was performed using the Vectashield mounting medium for fluorescence with DAPI kit from Vector Laboratories. Photomicrographs were taken from slides from xenotransplant tissues of HCT116 cells stained with DAPI. We acquired 30 photomicrographs per experimental group. The acquisition of images was done through an Olympus IX71 inverted microscope using the software Qcaturepro 5 of the company QImaging by means of the U-mwu2 filter, with an excitation band of 330–420 nm, dichroic mirror 400, on a microscopic scale Fluorite plan 20× NA0.45.

### 4.10. Immunohistochemistry-PCNA

After removing the paraffin as previously described, the antigen was exposed for which the samples were heated to boiling in a 0.25 mM sodium citrate solution for 20 min, followed by washes of PBS for 5 min. 

To perform the blockade of the endogenous peroxidase the tissues were exposed to 3% H_2_O_2_ for 30 min and then washed with PBS. Permeabilization of the membrane was performed with a Triton x-100 0.5% solution for 30 min, followed by washes with PBS. The samples were previously delimited by area with a wax pencil, and blocking was performed with 5% albumin for 30 min. Primary antibody anti-PCNA antibodies (sc-25280, Santa Cruz Biotechnology, INC) were used for overnight incubation at 4 °C. Secondary antibody bound to biotinylated anti-mouse IgG for PCNA (GTX77315, GeneTex) were used for incubation during 60 min at 37 °C. Immunohistochemical signals were detected with diaminobenzidine (DAB) for 10 min. Counterstaining to differentiate marked nuclei was done with immune-reactive products or with hematoxylin; a hematoxylin stain was performed for three to five min in each tissue and it was then covered with Li_2_CO_3_, after which the assembly was mounted using EcoMount from Biocare Medical. Photomicrographs of the immunostaining were taken for PCNA. Images were acquired using an Olympus IX71 inverted microscope (with the 20× objective lens) and the software Qcapturepro 5 of the company QImaging. Color brown was indicative of a positive response to PCNA. The quantification of PCNA determined by the Fiji software (http://fiji.sc), and was calculated as the average of antigen-positive cells in 10 microscopic fields randomly selected from 3 xenotransplantation tissues per experimental group, having a total of 30 photomicrographs analyzed per group.

### 4.11. Poly (ADP-Ribose) Polymerase (PARP-1) Determination

After removing the paraffin and following the experimental sequence of tissue preparation previously described for immunohistochemistry, the samples were incubated overnight at 4 °C with the primary anti-PARP-1 antibody (sc-8007, Santa Cruz Biotechnology, INC); the samples were later incubated with the secondary antibody bound to biotinylated anti-mouse IgG for PARP-1 (GTX77315, GeneTex) during 60 min at 37 °C. Immunohistochemical signals were detected as previously described. EcoMount from Biocare Medical was used as a mounting medium once the samples were completely dry. Images were acquired using an Olympus IX71 inverted microscope (with the 20× objective lens) and the software Qcapturepro 5 of the company QImaging. PARP-1 positive and claved-PARP-1 cells were quantified using the fiji.sc software. 

### 4.12. Western Blot

Mouse-monoclonal antibody β and Actin antibody (1:5000) were used as primary antibodies and revealed at 18 s of exposure. Mouse-monoclonal antibody p21 (1:100) was also used and revealed at 34 s of exposure. Antibodies were acquired from Santa Cruz Biotechnology (sc-47778 and sc-6246, respectively). The procedure was performed following the previously reported protocol [34]. Protein was extracted from the tumors, for which the tumors were crushed with an extraction buffer: 200 μL protease inhibitor, 800 μL lysis buffer, and PMSF 10 μL phosphatase inhibitor. After homogenization, they were centrifuged for 30 min at 4 °C, RFC 8000. The protein content was evaluated in the supernatant. The total protein content was determined using the Thermo Scientific Pierce BCA Protein Assay Kit (catalog number 23225) in 96-well plates at 570 nm. The samples were heated at 95 °C for 15 min in a thermocycler for protein denaturation. A total of 40 μg of protein was separated on an SDS page. Afterwards, the gels were transferred to polyvinylidene difluoride (PVDF) membranes (0.2 μM pore size). After the electrotransfer, the membranes were blocked with 5% BSA in TBST for 1 h. Subsequently, the primary antibody (mouse-monoclonal antibody p21) in 5% bovine serum albumin (BSA) was added and incubated overnight at 4 °C on a shaker. The next day the membrane was washed with TBST for 5 min for 3 times under stirring, and the secondary antibody was added and incubated for 1 h. The membrane was then washed with TBST for 5 min for 2 times and once again with TBS without Tween. Signal was development in the dark room.

### 4.13. Data Analysis

Each experiment was performed in triplicate. Cytotoxic activity, cytochemical staining, tumor volume, body weight and values of biochemical parameters are reported as mean ± standard deviation (SD) of three independent experiments. The data analysis for western blot was performed using Biorad’s Image Lab 6.0.1 program, which allowed us to obtain the numbers to determine the relative expression of the bands. 

Statistical analyses were performed using the GraphPad Prism 6.0 software (GraphPad Software Inc., La Jolla, USA). Significant difference between groups were determined by means of *t*-test and two-way ANOVA. *p*-Values less than 0.05 were considered as statistically significant. 

## 5. Conclusions

Choosing the correct dose and frequency of treatment is fundamental when seeking to determine the use and activity of potential naturally-occurring antitumor agents. Our results show that the administration of Argentatin A (AA) at a dose of 250 mg/kg three times a week for three weeks (21 days), decreases the tumor mass under the same administration scheme of cisplatin at a dose of 2 mg/kg. However, AA did not induce weight loss or liver damage as cisplatin did. Although more studies are required to determine the mechanism by which AA activates apoptosis, we propose that it could either become a chemotherapeutic agent, or could be used as a potential adjuvant in chemotherapy in order to reduce the toxic effects of the new therapeutic approaches being currently developed for cancer treatment. As in any process of new drug development further preclinical and lab studies will need to be performed to test the acute toxicity, biodistribution and pharmacokinetics of AA before the efficacy and security of this promising natural antitumor agent can be tested in clinical trials with humans. Notwithstanding the long road ahead, based on our results we certainly believe that AA could aid to trigger new cancer therapies, adding up major health benefits.

## 6. Patents

An application for an invention patent has been filed before the Mexican Institute of Industrial Property. Application number: MX/a/2019/006749.

## Figures and Tables

**Table 1:** Evaluation of the cytotoxic activity of AA in cells at different time points. Cisplatin was used as a positive control.

Incubation Time (h)		Compound IC_50_ (μM) ± SD.						
	AA				Cisplatin			
	Cancer Cell Lines				Cancer Cell Lines			
	HCT15	HCT116	SW620	HaCaT	HCT15	HCT116	SW620	HaCaT
24	95.43 ± 0.6	87.83 ± 0.7	115.62 ± 0.4	123.49 ± 0.6	12.50 ± 0.5	10.81 ± 0.7	18.03 ± 0.5	34.75 ± 0.5
48	59.67 ± 1.3	53.33 ± 0.2	72.46 ± 0.2	118.41 ± 1.2	10.72 ± 0.6	8.37 ± 0.5	13.87 ± 0.4	29.33 ± 0.6
72	44.83 ± 0.9	43.17 ± 0.4	61.33 ± 1.2	121.45 ± 1.3	4.68 ± 0.3	3.09 ± 0.3	9.09 ± 0.3	22.26 ± 0.4 ^1^

^1^ The values presented are the average of 3 independent experiments performed in triplicate each ± SD.

**Table 2:** Evaluation in blood of preclinical systemic toxicities of AA after treatment.

Blood Parameters	Reference	AA 500 mg/kg (Once Weekly)	AA 250 mg/kg (3 Times a Week)	Cisplatin 2mg/kg (3 Times a Week)	Cisplatin 4 mg/kg (Once Weekly)
Leukocytes	3.2–7.0 × 10^9^/L	6.5 × 10^9^/L	7.2 × 10^9^/L	1.6 × 10^9^/L ****	2.1 × 10^9^/L ****
Lymphocytes	3.16–7.8 × 10^9^/L	6.9 × 10^9^/L	7.3 × 10^9^/L	0.52 × 10^9^/L ****	0.52 × 10^9^/L ****
Erythrocytes	7.1–10.2 × 10^12^/L	7.29 × 10^12^/L	7.4 × 10^12^/L	7.14 × 10^12^/L	7.36 × 10^12^/L
Hemoglobin	149–170 g/L	158 g/L	164 g/L	113 g/L ****	115 g/L ****
Glucose	6.6–8.5 mmol/L	7.1 mmol/L	8.0 mmol/L	7.80 mmol/L	8.56 mmol/L
Urea	2.6–3.5 mmol/L	3.2 mmol/L	2.9 mmol/L	9.06 mmol/L ****	7.57 mmol/L ****
Creatinine	8.8–26.5 µM/L	20.16 µM/L	14.2 µM/L	43.3 µM/L ****	29.06 µM/L ****
Alanine Transferase	46–55 UL	49.6 UL	53.3 UL	75.21 UL ****	62.89 UL ****
Aspartate transferase	85–101 UL	98.96 UL	91.07 UL	197.32 UL ****	151.25 UL ****

The values of the biochemical parameters are reported as the mean ± SD of three mice per group. Significant difference **** *p* < 0.0001 compared to the reference values (*t* test).
